# Supplementary material for: A nanoluciferase SARS-CoV-2 for rapid neutralization testing and screening of anti-infective drugs for COVID-19
Source: Nat Commun. 2020 Oct 15;11:5214. doi: 10.1038/s41467-020-19055-7 (PMC7567097; doi:10.1038/s41467-020-19055-7)
Supplement: Supplementary file 1 — Supplementary information [file 41467_2020_19055_MOESM1_ESM.pdf]

Supplementary Information for "A nanoluciferase SARS-CoV-2 for rapid neutralization testing and screening of anti-infective drugs for COVID-19" by Xie et al.

Primer name	Sequences (5′->3′)
X83	GTTCGTTTAATCAATCTCCATTGG
X84	GAACTTTCATTAATTGACTTCTATTTG
X87	CCAATGGAGATTGATTAAACGAACATGGTCTTCACACTCGAAGATTTC
X88	CAAATAGAAGTCAATTAATGAAAGTTCTTAATTAATTACGCCAGAATGC GTTCGCACAG
X109	CATTATACGAAGTTATATTCGATGCGGCCGCGTCTCACACTAGCCATC CTTACTGCGC
X112	GTCGACTCTAGAGGATCCCACATCGATACGTCTCGTTTTTTT
Cov-T7-N-F	TACTGTAATACGACTCACTATAGGATGTCTGATAATGGACCCCAAAAT C
polyT-N-R	TTTTTTTTTTTTTTTTTTTTTTTTTTTTTTTTTTTTTAGGCCTGAGTTGAGT CAGCAC

Supplementary Table 1. Primers for construction of the subclone pCC1-F7-Nluc and amplifying N gene.

Primer Name	Sequences (5′->3′)
cov-1V	ATTAAAGGTTTATACCTTCCCAGG
cov-655V	AGCTGGTGGCCATAGTTAC
cov-1321V	AGGTGCCACTACTTGTGG
cov-1925V	CTGCTCAAAATTCTGTGCG
cov-2572V	CTACTAGTGAAGCTGTTGAAGC
cov-3225V	CTGTTGGTCAACAAGACGG
cov-3824V	GTTTCAAGCTTTTTGGAAATG
cov-4431V	TGCCTGTCTGTGTGGAAAC
cov-4990V	CAACATTAACCTCCACACGC
cov-5525V	ACTTGTGGACAACAGCAG
cov-6109V	GAAACCTGCTTCAAGAGAG
cov-6737V	ACACGGTGTTTAAACCGTG
cov-7382V	CAAATGGCCCCGATTTCAG
cov-7930V	TCAGCGTCTGTTTACTACAG
cov-8481V	CTTTTAAGTTGACATGTGCAAC
cov-8995V	ATCAGCTTGTGTTTTGGC
cov-9534V	CTGTACTCTGTTTAACACC
cov-10094V	GAGGGTTGTATGGTACAAG
cov-10680V	ACGCTGCTGTTATAAATGG
cov-11188V	ACCTTCTCTTGCCACTG
cov-11707V	AGTTTCTACACAGGAGTTTAG
cov-12205V	GAAGAAGTCTTTGAATGTGG
cov-12806V	GTACTTGCACTGTTATCCG
cov-13441V	GTCAGCTGATGCACAATCG
cov-14062V	GATAATCAAGATCTCAATGG
cov-14618V	CTACGTGCTTTTCAGTAG
cov-15170V	ATCAATAGCCGCCACTAG
cov-15677V	ACGCATATTTGCGTAAAC
cov-16273V	TCATTAAGATGTGGTGCTTG
cov-16853V	GTGATGCTGTTGTTTACCG
cov-17444V	CTCAATTACCTGCACCAC
cov-18037V	AAGCTGAAAATGTAACAGG
cov-18588V	TGTCTTATGGGCACATGG
cov-19211V	GATATCCTGCTAATTCCATTG
cov-19840V	ATTTGGGTGTGGACATTG
cov-20459V	AACAGATGCGCAAACAGG
cov-20934V	TACGCTGCTTGTCGATTC
cov-21521V	TGTTATTTCTAGTGATGTTCTTG
cov-22092V	TGGACCTTGAAGGAAAAC
cov-22685V	TCCACTTTTAAGTGTTATGGAG
cov-23203V	AGGCACAGGTGTTCTTAC
cov-23840V	GTACACAATTAAACCGTGC
cov-24428V	CACAAGCTTTAAACACGC
cov-25068V	TCTCTGGCATTAATGCTTC
cov-25624V	CACTTTGTTTGCAACTTGC
cov-26245V	CATTCGTTTCGGAAGAGAC
cov-26778V	GTCTTGTAGGCTTGATGTG
cov-27372V	ATGGAGATTGATTAAACGAAC
cov-27875V	TTGTCACGCCTAAACGAAC
cov-28994V	CAACAAGGCCAAACTGTC

cov-29611V	GTGCAGAATGAATTCTCG
cov-28404V	GTTTACCCAATAATACTGCG
pCC1-F	CGACAGGTGCTGAAAGCGAGC
M13F	GTAAAACGACGGCCAG
M13R	CAGGAAACAGCTATGAC

Supplementary Table 2. Primers for sequencing the seven subclones.

a

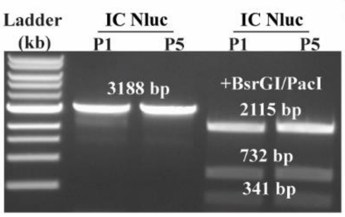

b

Genome position	IC WT	P5 IC Nluc	Amino acid change
2168	A	T	F635I, ORF1
22205	G	C	D215H, S
26960	T	C	R146R, M
28599	G	T	G96C, N
29292	G	A	D340N, N
27394-27761	ORF7a	ORFNluc	--

Supplementary Figure 1. **Stability of SARS-CoV-2-Nluc on A549-ACE2 cells.** (a) Restriction enzyme digestion of RT-PCR products. SARS-CoV-2-Nluc viruses were passaged five times on A549-ACE2 cells. The RT-PCR products (nucleotides 25,068-28,099 of viral genome) from the P1 and P5 viral RNA and their digestion products with BsrGI and PacI are shown. (b) Summary of full-genome sequences of P1 and P5 IC Nluc viruses on A549-ACE2 cells. Nucleotide and amino acid differences from the IC WT are indicated.
