# Supplementary material for: A nanoluciferase SARS-CoV-2 for rapid neutralization testing and screening of anti-infective drugs for COVID-19
Source: Nat Commun. 2020 Oct 15;11:5214. doi: 10.1038/s41467-020-19055-7 (PMC7567097; doi:10.1038/s41467-020-19055-7)
Supplement: Supplementary file 3 — Reporting Summary [file 41467_2020_19055_MOESM3_ESM.pdf]

nature research

Corresponding author(s): Pei-Yong Shi

Last updated by author(s): 8/24/2020

Reporting Summary

Nature Research wishes to improve the reproducibility of the work that we publish. This form provides structure for consistency and transparency in reporting. For further information on Nature Research policies, see our Editorial Policies and the Editorial Policy Checklist.

Statistics

For all statistical analyses, confirm that the following items are present in the figure legend, table legend, main text, or Methods section.

n/a	Confirmed	
☐	☒	The exact sample size (n) for each experimental group/condition, given as a discrete number and unit of measurement
☐	☒	A statement on whether measurements were taken from distinct samples or whether the same sample was measured repeatedly
☐	☒	The statistical test(s) used AND whether they are one- or two-sided<br>*Only common tests should be described solely by name; describe more complex techniques in the Methods section.*
☐	☒	A description of all covariates tested
☐	☒	A description of any assumptions or corrections, such as tests of normality and adjustment for multiple comparisons
☐	☒	A full description of the statistical parameters including central tendency (e.g. means) or other basic estimates (e.g. regression coefficient) AND variation (e.g. standard deviation) or associated estimates of uncertainty (e.g. confidence intervals)
☐	☒	For null hypothesis testing, the test statistic (e.g. F, t, r) with confidence intervals, effect sizes, degrees of freedom and P value noted<br>*Give P values as exact values whenever suitable.*
☒	☐	For Bayesian analysis, information on the choice of priors and Markov chain Monte Carlo settings
☒	☐	For hierarchical and complex designs, identification of the appropriate level for tests and full reporting of outcomes
☐	☒	Estimates of effect sizes (e.g. Cohen's d, Pearson's r), indicating how they were calculated

Our web collection on statistics for biologists contains articles on many of the points above.

Software and code

Policy information about availability of computer code

Data collection	not applicable
Data analysis	not applicable

For manuscripts utilizing custom algorithms or software that are central to the research but not yet described in published literature, software must be made available to editors and reviewers. We strongly encourage code deposition in a community repository (e.g. GitHub). See the Nature Research guidelines for submitting code & software for further information.

Data

Policy information about availability of data

All manuscripts must include a data availability statement. This statement should provide the following information, where applicable:

- Accession codes, unique identifiers, or web links for publicly available datasets
- A list of figures that have associated raw data
- A description of any restrictions on data availability

All data supporting the findings in this study are detailed in the paper. Source data are provided within this paper. The nanoluciferase SARS-CoV-2 is available from the World Reference Center for Emerging Viruses and Arboviruses (WRCEVA) at the University of Texas Medical Branch (UTMB) (https://www.utmb.edu/wrceva). The reagent can be used for research without any constraints. If used for commercial or profit purpose, please contact UTMB's Technology Office.

Field-specific reporting

Please select the one below that is the best fit for your research. If you are not sure, read the appropriate sections before making your selection.

☒ Life sciences ☐ Behavioural & social sciences ☐ Ecological, evolutionary & environmental sciences

For a reference copy of the document with all sections, see nature.com/documents/nr-reporting-summary-flat.pdf

Life sciences study design

All studies must disclose on these points even when the disclosure is negative.

Sample size	not applicable
Data exclusions	All data are included in the study.
Replication	Experiments were performed in duplication and repeated at least once.
Randomization	not applicable
Blinding	not applicable

Reporting for specific materials, systems and methods

We require information from authors about some types of materials, experimental systems and methods used in many studies. Here, indicate whether each material, system or method listed is relevant to your study. If you are not sure if a list item applies to your research, read the appropriate section before selecting a response.

Materials & experimental systems

n/a	Involved in the study	
☐	☒	Antibodies
☐	☒	Eukaryotic cell lines
☒	☐	Palaeontology and archaeology
☒	☐	Animals and other organisms
☐	☒	Human research participants
☒	☐	Clinical data
☒	☐	Dual use research of concern

Methods

n/a	Involved in the study	
☒	☐	ChIP-seq
☒	☐	Flow cytometry
☒	☐	MRI-based neuroimaging

Antibodies

Antibodies used	anti-hACE2, Cat# AF933, lot HOK0320032, supplier R&D systems; anti-hDPPIV/CD26, Cat# AF1180, lot JOQ021901, supplier R&D systems;
Validation	anti-hACE2 antibody detects human ACE-2 in direct ELISAs and Western blots. In direct ELISAs and Western blots, less than 1% cross-reactivity with recombinant human ACE is observed. The antibody has applications in Westenr blot, immunohistochemistry and immunoprecipitation, Simple Western, blockage of receptor-ligand interaction (citation: Hofffman, M. et al. (2020) cell. DOI: 10. 1016/j.cell. 2020.02.052). Reference: https://www.rndsystems.com/products/human-ace-2-antibody_af933.<br>anti-hDPPIV/CD26 detects human DPPIV/CD26 in direct ELISAs and Western blots. In direct ELISAs, less than 5% cross-reactivity with recombinant mouse DPPIV is observed. The antibody has applications in Westenr blot, immunohistochemistry and immunoprecipitation, Simple Western, immunocytochemistry. Reference: https://www.rndsystems.com/products/human-dppiv-cd26-antibody_af1180

Eukaryotic cell lines

Policy information about cell lines

Cell line source(s)	Vero (ATCC®CCL-81) and Vero E6 (ATCC® CRL-1586), A549 (ATCC® CCL-185™) were purchased from the American Type Culture Collection (ATCC, Bethesda, MD). A549-ACE2 cells were previously established in UTMB using a pseudotyped retrovirus that expressing human ACE2 (doi:10.1128/JVI.79.6.3846–3850.2005).
Authentication	ATCC have comprehensively performed authentication on Vero, Vero E6 cell lines, A549 cells. A549-ACE2 cells were established in UTMB and has been validated for SARS infection.
Mycoplasma contamination	All cell lines were tested negative for mycoplasma.

Commonly misidentified lines (See ICLAC register)	not applicable

Human research participants

Policy information about studies involving human research participants

Population characteristics	Human serum samples were the leftover samples in EDTA/SST tubes received by UTMB for the routine diagnostic purposes. No patient identifying information was recorded in this study – all samples were de-identified. There are no concerns with patient privacy.
Recruitment	The research protocol regarding the use of human serum specimens was reviewed and approved by the University of Texas Medical Branch (UTMB) Institutional Review Board. The approved IRB protocol number is 20-0070. All human serum specimens were obtained at the UTMB. All specimens were de-identified from patient information. No consent of the use of patient serum samples were required for this study.
Ethics oversight	The research protocol has been approved by the UTMB Institutional Review Board (IRB number 20-0070). We have included this IRB protocol information in the manuscript.

Note that full information on the approval of the study protocol must also be provided in the manuscript.
